# The pathogenesis mechanism and potential clinical value of lncRNA in gliomas

**DOI:** 10.1007/s12672-024-01144-4

**Published:** 2024-07-05

**Authors:** Yuan Liu, Hui Yuan, JingJia Fan, Han Wang, HuiYu Xie, JunFeng Wan, XueYing Hu, Jie Zhou, Liang Liu

**Affiliations:** 1 Department of Clinical Medicine, School of Clinical Medical, Southwest Medical University, Luzhou, 646000 Sichuan China; 2 Dept Neurosurg, Affiliated Hosp, Southwest Med Univ, Luzhou, 646000 People’s Republic of China

**Keywords:** Glioma, lncRNA, Biological function, Therapy, Diagnosis, Prognosis

## Abstract

Glioma is the most common malignant tumor in the central nervous system, and its unique pathogenesis often leads to poor treatment outcomes and prognosis. In 2021, the World Health Organization (WHO) divided gliomas into five categories based on their histological characteristics and molecular changes. Non-coding RNA is a type of RNA that does not encode proteins but can exert biological functions at the RNA level, and long non-coding RNA (lncRNA) is a type of non-coding RNA with a length exceeding 200 nt. It is controlled by various transcription factors and plays an indispensable role in the regulatory processes in various cells. Numerous studies have confirmed that the dysregulation of lncRNA is critical in the pathogenesis, progression, and malignancy of gliomas. Therefore, this article reviews the proliferation, apoptosis, invasion, migration, angiogenesis, immune regulation, glycolysis, stemness, and drug resistance changes caused by the dysregulation of lncRNA in gliomas, and summarizes their potential clinical significance in gliomas.

## Introduction

Glioma is the most invasive primary intracranial tumor, occurring in all age groups but more commonly in adults, with males being more susceptible than females [1, 2]. Among them, glioblastoma is the most deadly brain tumor, with a median survival time of only 12–15 months for patients with glioblastoma multiforme (GBM) and a 5-year survival rate of only 3–5% [3–5]. Due to the high malignancy of gliomas, although surgical and drug treatments have made some progress, the cure rate is low, prognosis is poor, and recurrence rate is high. Therefore, it is urgently necessary to clarify the pathological mechanisms of gliomas to improve diagnostic accuracy and develop targeted therapeutic drugs.

Long non-coding RNA (LncRNA) is a type of non-coding RNA longer than 200 nt, mainly responsible for gene regulation, including regulation before transcription or after translation [6]. These LncRNAs not only participate in basic life processes such as growth, differentiation, and development, but also play an essential role in processes such as proliferation, apoptosis, invasion, and angiogenesis in various diseases, including cancers, making their study in diseases of significant potential significance. These physiological processes provide important directions for subsequent diagnosis, treatment, and prognosis [7]. Therefore, studying the mechanisms of LncRNA provides new insights for finding new therapeutic targets and developing new drugs.

This article provides an overview of the occurrence, development, and roles of lncRNA in glioma. It introduces the proliferation and apoptosis, invasion and migration, angiogenesis, immune regulation, glycolysis, stemness, and drug resistance in glioma. It also summarizes the potential clinical significance of lncRNA in glioma, including future diagnosis, treatment, and prognosis assessment.

## The current research status of glioma

Glioma originates from glial cells and is one of the most common malignant tumors in the brain, accounting for about 80% of all malignant tumors in the central nervous system (CNS) [8]. The World Health Organization classifies glioma into four levels of malignancy, with levels 1 and 2 referred to as low-grade glioma (LGG), and levels 3 and 4 referred to as high-grade glioma (HGG). Among level 4 glioma, the most common type is GBM [9–11]. In the 5th edition of the WHO classification of CNS tumors in 2021, clinical and pathological criteria are closely integrated with molecular subtypes, emphasizing the role of molecular changes in CNS tumor classification. The new classification proposed in the 5th edition separates gliomas into the following five categories (Table 1) [3]. From the above, it can be seen that gliomas in the 5th edition classification distinguish between tumors occurring in adults and children, and classify them into diffuse glioma and localized glioma based on their growth and infiltration patterns. However, the diagnostic classification of adult-type diffuse glioma and pediatric-type diffuse glioma is not solely based on age, but also on major molecular variations and the distribution characteristics of tumors in different age groups [12]. The mutation of isocitrate dehydrogenase (IDH) is an important molecular feature of adult-type diffuse glioma; MYB/MYBL1 variations and mitogen-activated protein kinase (MAPK) signaling pathway variations are important molecular features of pediatric-type diffuse low-grade glioma; histone H3 variations are the main molecular features of pediatric-type diffuse high-grade glioma [13, 14]. Adult-type diffuse glioma is the main type in adults, but can also occur in children; pediatric-type diffuse glioma mainly occurs in children but can also occur in adults, especially in young people [15].

**Table 1:** The latest classification of glioma

WHO classification
Adult-type diffuse glioma
Pediatric-type diffuse low-grade glioma
Pediatric-type diffuse high-grade glioma
Localized astrocytoma
Ependymal tumor

The current treatment methods for gliomas mainly include: surgical treatment, radiotherapy, chemotherapy, immunotherapy, etc. [16]. The standard treatment for malignant gliomas is maximal safe surgical resection combined with postoperative radiotherapy and chemotherapy [17]. Due to the invasive growth characteristics of malignant gliomas, complete resection is rarely achieved [18]. Radiotherapy is an important supplement to glioma treatment. Temozolomide is the most common and effective chemotherapy drug for malignant glioma patients, but the efficacy of alkylating agents such as TMZ is related to the methylation status of the O6-methylguanine-DNA methyltransferase (MGMT) promoter in gliomas. Patients with non-methylated MGMT promoters are more likely to develop alkylating agent resistance [19]. For patients with GBM, the outcome is almost always progression or recurrence [20]. One of the reasons for the poor prognosis of GBM patients is the tumor’s resistance to chemotherapy and radiotherapy, as well as insufficient understanding of the resistance and migration mechanisms of tumors, leading to such a high recurrence and progression rates [21]. With continuous exploration of lncRNA and in-depth study of its biological functions, it provides new insights for us to understand the occurrence, development, diagnosis, treatment, and prognosis of gliomas.

## Long non-coding RNA involvement in the pathogenic mechanisms of glioma

### The characteristics of lncRNA

Non-coding RNA is a class of RNA molecules that do not encode proteins but can exert their biological functions at the RNA level. Whole-genome RNA sequencing shows that over 90% of the human genome is transcribed, with only about 3% containing protein-coding genes. The remaining portion of the genome mainly encodes non-coding RNA, which is responsible for maintaining the stability of protein expression in cells [22]. lncRNA is a type of non-coding RNA with a length ranging from 200 to 100,000 nucleotides. It is typically transcribed by RNA polymerase II and controlled by various transcription factors. lncRNA plays an indispensable role in various regulatory processes within cells, including cell differentiation, dosage compensation effects, epigenetic regulation, and cell cycle control [23, 24]. Based on their genomic locations, lncRNAs can be classified into different types, including antisense long non-coding RNA, intronic non-coding RNA, long intergenic non-coding RNA (lincRNA), promoter-associated lncRNA, and non-coding region lncRNA. Studies have shown that the transcription of lncRNAs can directly interfere with promoter activity or modify chromatin structure to positively or negatively influence downstream gene expression [25]. In the cell nucleus, lncRNAs collaboratively interact with chromatin transcription and modify newly synthesized RNA. In the cytoplasm, they regulate mRNA turnover, storage, translation, and coordinate protein processing events [26]. Compared to mRNA, lncRNAs often lack highly conserved sequences across species and are expressed at lower levels. However, lncRNAs display stronger tissue-specific expression patterns [27], which makes their study in diseases of significant potential significance.

According to previous research, it has been confirmed that the dysregulation of lncRNA plays a crucial role in the pathogenesis, progression, and malignancy of glioma [28, 29] For example, a lncRNA called PSMB8-AS1 can enhance the proliferation and migration of glioma cells, as well as inhibit apoptosis, by competitively binding with miR-382-3p and increasing the expression of branched-chain amino acid transferase 1 (BCAT1) [30]. Various lncRNAs show significant differences in expression levels between glioma and normal tissues, indicating their potential significance in the occurrence and development of glioma. Furthermore, abnormal expression of lncRNAs in clinical glioma specimens has been proven to be closely associated with malignant grading and histological differentiation [31], which holds important potential clinical implications for glioma. Tables 2 and 3 provides a comprehensive list of the latest research on oncogenic LncRNAs and tumor suppressor LncRNAs associated with glioblastoma, as well as their potential functional mechanisms.

**Table 2:** Oncogenic lncRNAs associated with Glioma

Name	Targets/regulator	Functional mechanism	Pathway	References
HOXA11-AS	let-7b-5p;Tpl2	Promoted the proliferation, migration, ROS resistance, and invasion of glioma cells	let-7b-5p/CTHRC1/β-catenin/c-Myc; c-Jun/Tp12/Tpl2-MEK1/2-ERK1/2	[34]
DDX11-AS1	HNRNPC	Promoted glioma cell proliferation and migration	Wnt/β-catenin; AKT; the epithelial-mesenchymal transition process	[35]
LUCAT1	ABCB1	Promote the proliferation and invasion of malignant glioma cells	LUCAT1/ABCB1/ROS	[36]
LIMD1-AS1	–	Promotes the proliferation, colony formation, migration and invasion of glioma cells, as well as the growth of xenograft tumors of glioma cells in vivo	–	[38]
CHASERR	miR-6893-3p	Promotes the proliferation, migration and invasion of glioma	m6A/lncRNACHASERR/miR-6893-3p/TRIM14	[40]
LINC01088	SNRPA	Promotes the growth and invasion of glioma cells	–	[44]
ANCR	EZH2;PTEN	Promotes glioma cells invasion, migration, proliferation and inhibits apoptosis	–	[47]
WEE2-AS1	miR-29b-2-5p	Promotes the proliferation, migration and invasion of glioma cells	Lnc RNA WEE2-AS1/miR-29b-2-5p/TPM3	[48]
FOXD3-AS1	miR-128-3p;miR-128-3p	Promotes the proliferation, migration, invasion, angiogenesis and temozolomide resistance of glioma cells	FOXD3-AS1/miR-128-3p/IGF2BP3;miR-128-3p/WEE1 G2 checkpoint kinase	[52, 90]
H19	miR-342; miR-138; KSRP	Promotes cell proliferation, migration, angiogenesis and resistance to TMZ of Glioma	LncRNA H19/miR-342/Wnt5a/β-catenin; LncRNA H19/miR-138/HIF-1α	[55, 56]
PAXIP1-AS1	ETS1	Promotes the invasion and angiogenesis of glioma cells	LncRNA PAXIP1-AS1/ETS1/KIF14	[54]
LINC00346	–	Promotes the Angiogenesis of Glioma	ANKHD1/LINC00346/Staufen1/ZNF655	[53]
HULC	–	Stimulates the epithelial-mesenchymal transition process and vasculogenic mimicry in human glioblastoma	–	[58, 59]
ALKBH5	–	Promoted proliferation, migration, and invasion of glioma cells and recruited the M2 macrophage to glioma cells	–	[64]
LINC01271	–	Increases the malignancy of tumors by enhancing the abundance of M1 subtype macrophages in the immune microenvironment	–	[65]
CRNDE	–	Promotes the occurrence and development of glioma by altering tumor immunity and weakens temozolomide chemosensitivity	–	[66, 92]
LINC00346	–	Promoting proliferation, migration, and immune infiltration of glioma	–	[67]
NEAT1	PGK1	Promotes the proliferation and glycolysis of glioma cells	–	[71]
LINC00689	miR-338-3p	Promotes the proliferation, migration, invasion, and glycolysis of glioma cells	LINC00689/miR-338-3p/PKM1	[72]
SNHG14	IRF6	Promotes the proliferation and glycolysis of glioma cells	Lin28A/SNHG14/IRF6	[73]
LINC00174	miR-152-3p/SLC2A1	Promotes the proliferation, migration, invasion, and glycolysis of glioma cells	miR-152-3p/SLC2A1	[74]
DUXAP10	HuR	Significantly enhances the stemness of glioma cells	HuR/Sox12	[78]
HULC	FOXM1	Increases the stemness of glioma cells, promotes their proliferation, and induces apoptosis and differentiation of GSCs	FOXM1/AGR2/HIF-1α	[79]
SNHG10	miR-532-3p	Promotes the proliferation, migration, invasion, and stemness of glioblastoma cells	SNHG10/miR-532-3p/FBXL19	[80]
BC200	miR-218-5p	Promotes the proliferation, self-renewal, pluripotency, and temozolomide (TMZ) chemoresistance of GB cells	BC200/miR218-5p	[82]
FOXD2-AS1	TAF-1	Promotes the stemness and proliferation of GSCs, and inhibits their apoptosis and differentiation	FOXD2-AS1/TAF-1/NOTCH1	[83]
LINC00265	miR-let-7d-5p;IGF2BP2	Promotes the stemness and EMT of glioma cells	LINC00265/miR-let-7d-5p/IGF2BP2/ZNF384/IFI30	[84]
MUF	SMAD2/3;TGF	Enhances GBM resistance to TMZ	–	[86]
HOXD-AS2	STAT3;KSRP	Weakens the sensitivity of glioblastoma to TMZ	HOXD-AS2-STAT3 feedback loop	[87, 88]
PDIA3P1	C/EBPβ;MDM2	Promotes Temozolomide resistance in glioblastoma	–	[76]
RMRP	–	Increases temozolomide resistance in glioma	LncRNA-RMRP/ZNRF3 axis and Wnt/β-catenin signaling	[91]
DANCR	FOXO1	Increases glioblastoma Cell Chemoresistance	DANCR/FOXO1/PID1	[93]

**Table 3:** Tumor-suppressive lncRNAs associated with glioma

Name	Targets/regulator	Functional mechanism	Pathway	References
LINC01018	miR-942-5p	Inhibits the migration, invasion, and proliferation of glioma cells	LncRNA LINC01018/miR-942-5p/KNG1	[41]
TUSC7	miR-10a-5p	Inhibit the proliferation and migration of glioma cells	miR-10a-5p;BDNF/ERK	[42]
CACNA1C-AS2	FBX045	Suppressed growth, migration and invasion of glioma cells	CACNA1C-AS2/Fbxo45/mTOR	[43]
SLC26A4-AS1	NFKB1	Inhibit the Angiogenesis of Glioma	LncRNA SLC26A4-AS1/NPTX1/NFKB1	[57]
ZBED3-AS1	SPI1	Inhibits TMZ resistance, vitality, migration, and glycolytic activity of TMZ-resistant cells	SPI1/THBD	[75, 89]
GAS5	SPACA6	Overexpression reduces cell viability and inhibits the migration and invasion of GBM cells	SPACA6 /miR-125a/miR-let-7e	[81]

### The regulation of lncRNAs in glioma proliferation and apoptosis

Multiple studies have shown that lncRNAs can impact cell cycle checkpoints, senescence, and apoptosis pathways through various signaling pathways such as the p53 pathway, receptor tyrosine kinase pathways, and the phosphoinositide 3-kinase (PI3K) pathway, leading to dysregulated cell death and enhanced cell viability [32, 33]. For example, in gliomas, lncRNA HOXA11-AS is highly expressed, indicating a poor prognosis. HOXA11-AS sequesters let-7b-5p in the cytoplasm, antagonizing its ability to suppress CTHRC1 expression, thereby activating the β-catenin/c-Myc pathway and promoting glioma cell proliferation in vitro and in vivo [34]. Another study found significant upregulation of lncRNA DDX11-AS1 in glioma tissues, and its overexpression may indicate a poor prognosis. Functional studies have demonstrated that DDX11-AS1 interacts with RNA-binding protein heterogeneous nuclear ribonucleoprotein C (HNRNPC), promoting Wnt/β-catenin and AKT pathways, as well as epithelial-mesenchymal transition, thereby facilitating glioma cell proliferation [35]. Abnormal expression of lung cancer associated transcript 1 (lncRNA LUCAT1) can affect glioma cell proliferation by regulating ABCB1 and promoting the activation of the RAS signaling pathway [36].

Upstream transcripts and enhancer-associated RNAs transcribed from promoters or enhancers share similarities in their functional mechanisms and regulation of DNA elements [37]. For example, Chen et al. [38] demonstrated through RT-qPCR analysis that lncRNA LIMD1-AS1 is significantly upregulated in glioma compared to normal brain tissue. Activation of CDK7 significantly enhances the recruitment of MED1 to the super-enhancer of LIMD1-AS1, thereby enhancing the expression of LIMD1-AS1 and promoting glioma cell proliferation. Many glioma-related lncRNAs act as miRNA sponges, reducing the availability of transcriptional miRNAs and leading to increased expression of their downstream target genes [39]. For instance, a study showed that lncRNA CHASERR is significantly upregulated in glioma tissues and indicates a poor prognosis in glioma patients. lncRNA CHASERR sequesters miR-6893-3p and positively regulates TRIM14 expression, thereby promoting glioma growth. The interplay among lncRNA CHASERR, miR-6893-3p, and TRIM14 can activate the PTEN/p-Akt/mTOR and Wnt/β-catenin pathways to regulate glioma growth [40]. Xu et al. [41] demonstrated through experiments that the overexpression of lncRNA linc01018 can inhibit the proliferation of mouse glioma cells and the growth of glioma by targeting the miR-942-5P/KNG1 axis. Quantitative polymerase chain reaction (qPCR) revealed significant upregulation of miR-10a-5p in glioma cells, which showed a negative correlation with TUSC7 expression. Dual-luciferase reporter gene assays further confirmed that overexpressed TUSC7 can bind to and significantly inhibit miR-10a-5p expression, thereby suppressing human glioma cell proliferation. TUSC7 can also regulate cell cycle and cyclin expression through the brain-derived neurotrophic factor/extracellular signal-regulated kinase (BDNF/ERK) pathway [42].

Another study found that a partial reverse complementary lncRNA called CACNA1C-AS2 inhibits the growth of glioma cells by negatively regulating the expression of Fbxo45 in glioma through rescue experiments [43]. Peng et al. [44] discovered that in glioma tissues and cell lines, linc01088 is highly expressed, and the level of linc01088 is positively correlated with SNRPA levels. Further rescue experiments confirmed that linc01088 can physically interact with Small Nuclear Ribonucleoprotein Polypeptide A (SNRPA) and regulate the expression of SNRPA at the transcriptional level, thereby inducing the growth of glioma cells.

### The role of lncRNAs in the invasion and migration of glioma

Infiltrative growth is the most prominent pathological characteristic of gliomas, primarily observed in critical structures such as white matter tracts and perivascular spaces. Gliomas can invade and damage adjacent and surrounding tissues, which is the main cause of treatment failure [45, 46]. Numerous studies have demonstrated that lncRNAs can promote the invasive and migratory abilities of gliomas, participating in glioma development. For example, a research has found that TGF-β is highly expressed in GBM, promoting the expression of LINC01711 in GBM tissue, thus enhancing the proliferation, migration, and invasion of GBM. Furthermore, experimental evidence has demonstrated that LINC01711 functions as a competitive endogenous RNA for 47miR-34a, promoting ZEB1 expression to regulate invasion [47]. Another study has found that the expression of lncRNA HOXA11-AS is positively correlated with the grading of glioma patients, with higher levels observed in high-grade gliomas compared to LGG and normal tissues. In vitro and in vivo experiments have shown that ectopic overexpression of HOXA11-AS promotes glioma cell migration and invasion through the TNF-α pathway, with Tpl2 serving as a key mediator [34]. Cheng et al. [48] discovered that lncRNA Antisense Non-Coding RNA in the INK4 Locus (ANCR) is overexpressed along with zeste homolog 2 (EZH2) in glioma tissues and cell lines, while phosphatase and tension homolog (PTEN) is downregulated. ANCR can enhance the invasion and migration of glioma cells by interacting with EZH2 and regulating PTEN expression. Another study indicated that Knockdown of lncRNA WEE2-AS1 suppresses glioma cell migration and invasion by modulating the miR-29b-2-5p/TPM3 axis [49]. In vitro and in vivo experiments revealed that ncRNA linc01018 regulates the malignant progression of gliomas through the miR-942-5p/KNG1 axis, enhancing glioma cell migration and invasion while modulating the expression of metastasis-related genes. The prognosis of patients is frequently positively associated with low expression of miR-942-5p [41]. High levels of lncRNA LIMD1-AS1 are significantly correlated with poor prognosis in glioma patients. Overexpression of LIMD1-AS1 significantly enhances glioma cell migration and invasion, with its function being regulated by super-enhancer activation. Experimental evidence suggests that inhibiting CDK7 significantly mitigates the recruitment of MED1 to super-enhancers, providing a promising strategy for glioma treatment [38].

### The regulation of lncRNAs in glioma angiogenesis

Large amounts of research have shown that the formation of new blood vessels plays an essential role in tumor growth and metabolism. Therefore, angiogenesis is considered a hallmark of malignant tumor development and progression [50]. For gliomas, their characteristic is abundant angiogenesis, and their growth and metastasis depend on angiogenesis [51, 52]. Additionally, glioblastoma multiforme (IV-grade glioma) is the most invasive histological subtype of glioma, and excessive microvascular proliferation is one of its main pathological features [53]. It has been found that dysregulation of certain lncRNAs plays a key role in tumor angiogenesis [54]. For example, Zhao et al. [55] found that lncRNA FOXD3-AS1 acts as a sponge for miR-128-3p by upregulating IGF2BP3 in gliomas, promoting glioma angiogenesis. Through RT-qPCR, it was found that lncRNA H19 and Wnt5a are significantly upregulated in glioma tissues and cells, while miR-342 is downregulated. Further functional analysis confirmed that H19 can directly target miR-342 to promote the expression of Wnt5a and activate the β-catenin signaling pathway to promote angiogenesis in glioma [56]. Additionally, lncRNA H19 can also act as a ceRNA through the miR-138/HIF-1α axis to promote glioma angiogenesis [54]. Through verification experiments, it was found that lncRNA PAXIP1-AS1 is highly expressed in glioma tissues and cells, accompanied by upregulation of KIF14. Overexpression of lncRNA PAXIP1-AS1 enhances the migration, invasion, and angiogenesis of human umbilical vein endothelial cells in glioma by recruiting transcription factor ETS1 to upregulate KIF14 expression [57]. The ANKHD1 protein, which contains the ANK repeat motif and one KH domain, can bind and enhance the stability of lncRNA linc00346 and upregulate its expression. At the same time, linc00346 can lower the stability of ZNF655 and promote its mRNA degradation, resulting in low expression of zinc finger protein 655 (ZNF655) and weakened inhibition of glioma angiogenesis. In addition, ZNF655 directly binds to the ANKHD1 promoter region to inhibit its transcription, further forming an ANKHD1/LINC00346/ZNF655 feedback loop, which can regulate glioma angiogenesis through messenger RNA decay mediated by Staufen1 [52]. Li et al. [58] found that lncRNA SLC26A4-AS1 is downregulated in human glioma tissues and cells. Further research confirmed that the downregulation of SLC26A4-AS1 weakens the recruitment of NFKB1 and reduces the transcriptional activity of NPTX1, resulting in weakened anti-angiogenic effects. Additionally, studies have shown that lncRNA HULC is significantly upregulated in human GBM14. The upregulation of lncRNA-HULC may enhance tube formation and invasion of human GBM cells through increased expression of MMP2 and MMP9, and promote the growth of GBM tumors in mice, while silencing lncRNA-HULC has the opposite effect [59, 60].

### The role of lncRNAs in glioma immune regulation

A large number of studies have shown that lncRNAs play important roles in the immune response of tumors through various regulatory mechanisms. They may regulate the tumor immune microenvironment by modulating immune-related functions, thus altering the occurrence and development of tumors, as well as the nature of cell type-specific gene expression. There is also evidence supporting the view that lncRNAs exhibit different cellular functions in the tumor immune microenvironment (TIME) [61, 62]. Past studies have demonstrated that various lncRNAs can impact the immune response of glioma through their diverse functions [63, 64]. For example, ALKB homolog 5 (ALKBH5), a gene related to ALKB, is enriched in the immune signaling pathway of glioma. Its expression is associated with the expression of immune checkpoint genes and can recruit M2 macrophages to glioma cells, thus affecting the sensitivity to immunotherapy [65]. A PD-L1-related lncRNA, linc01271, can be used independently for prognosis evaluation in glioma patients. Overexpression of linc01271 is associated with poorer prognosis, and it leads to higher abundance of M1 subtype macrophages in the immune microenvironment [66]. Differential expression of colorectal neoplasia expressed (CRNDE) is positively correlated with tumor immunity, as it can increase the expression of immune checkpoints such as CD274 (PDL1), PDCD1LG2 (PD-L2), CD86, and CD276. Moreover, in LGG, CRNDE expression is positively correlated with HLA molecules and chemokines, serving as an adverse prognostic indicator for glioblastoma patients [67]. lncRNA linc00346, associated with inflammation, is highly expressed in HGG. Experimental evidence has shown that linc00346 is closely related to immune cells, including macrophages, neutrophils, and Th2 cells. Its expression is positively correlated with the expression of CD274 and PDCD1LG2 genes, while negatively correlated with the expression of lymphocyte activation gene 3 (LAG3). Additionally, linc00346 expression is highly positively correlated with the abundance of M2 macrophages, collectively influencing the grading of glioma. Therefore, linc00346 is an adverse prognostic marker for glioma but also provides a potential therapeutic target for glioma treatment [68].

### The regulation of lncRNAs in glioma glycolysis

Metabolism differs greatly between cancer cells and normal cells, and the Warburg effect is a characteristic feature of cancer cells. The Warburg effect refers to the metabolic reprogramming of cancer cells, where glycolysis becomes the main energy source to meet the biological energy, biosynthesis, and redox needs of tumor cells [69, 70]. Previous studies have shown that lncRNAs can change the relevant glycolysis of tumors through the interaction between lncRNAs and metabolic enzymes, transcription factors, and other proteins involved in metabolic pathway [71]. For example, research has shown that overexpression of the lncRNA NEAT1 significantly promotes glycolysis in glioma cells. NEAT1 specifically interacts with phosphoglycerate kinase 1 (PGK1) to promote PGK1 stability. Inhibition of NEAT1 significantly inhibits tumor growth in mice, but PGK1 can reverse this effect, indicating that NEAT1 overexpression promotes tumor development [72]. Compared to normal brain tissue, linc00689 is highly expressed in gliomas. It directly interacts with miR-338-3p to promote the expression of pyruvate kinase M2 (PKM2), functioning as a competitive endogenous RNA, thus promoting glycolysis in gliomas [73]. In gliomas, RNA-binding protein lin28A and lncRNA SNHG14 are upregulated, while the transcription factor IRF6 is downregulated. Knocking out lin28A in cells decreases SNHG14 stability and expression, leading to degradation inhibition of IRF6 mRNA mediated by targeting gene interferon regulatory factor 6 (IRF6)‘s 3’ untranslated region and inhibiting STAU1, resulting in increased expression of IRF6. IRF6 inhibits the transcription of PKM2 and GLUT1, impairing glycolysis in gliomas. Therefore, the lin28A/SNHG14/IRF6 axis is crucial for reprogramming glucose metabolism and promoting tumor development in glioma cells [74]. Overexpression of linc00174 in glioma tissue and cell lines inhibits glycolysis in glioma cells and plays a suppressing role in tumor development. Additionally, the inhibitory effect of knocking down linc00174 in glioma cells can be reversed by miR-152-3p inhibitor or overexpression of SLC2A1 [75]. Research has shown that overexpression of the lncRNA ZBED3-AS1 in TMZ-resistant GBM cells inhibits glycolytic activity in glioma cells. ZBED3-AS1 binds to the Spi-1 oncogene (SPI1), blocking the transcriptional activation of platelet regulatory protein (THBD) mediated by SPI1. Upregulation of SPI1 and THBD increases glycolysis in glioma cells. In vitro experiments have shown that overexpression of ZBED3-AS1 or knocking down SPI1 blocks the growth of xenograft tumors in nude mice. Therefore, lncRNA ZBED3-AS1 provides an important target for the treatment of gliomas [76].

### The regulation of lncRNAs in glioma stemness

Glioma stem cells (GSCs) are considered one of the fundamental causes for the occurrence and malignant progression of gliomas [77]. Numerous studies have demonstrated that lncRNAs play important roles in regulating the self-renewal and differentiation abilities of GSCs by modulating stemness-related genes and cancer stem cell (CSC) gene expression [78]. For instance, research has shown that lncRNA DUXAP10 is significantly upregulated in neural glioma tissues compared to surrounding tissues. Functional experiments have confirmed that lncRNA DUXAP10 directly interacts with HuR, inhibiting the cytoplasmic-to-nuclear translocation of HuR protein. Subsequently, HuR binds directly to Sox12, enhancing the stability of Sox12 mRNA and promoting the expression of Sox12. This ultimately increases the stemness of glioma cells [79]. Another study found that lncRNA HULC stabilizes the expression of FOXM1 in GSCs through ubiquitination. FOXM1 then activates the transcription of AGR2, which promotes the expression of HIF-1α, further maintaining the stemness of GSCs [80]. lncRNA SNHG10 was found to be significantly upregulated in glioma cell lines, and functional experiments revealed that SNHG10 sponge sequesters MiR-532-3p, leading to increased expression of downstream FBXL19. This further promotes the stemness of glioma cells [81]. lncRNA GAS5 was found to be downregulated in high-grade glioma tissues and cells. Experimental evidence confirmed that GAS5 promotes the expression of SPACA6 by reducing its degradation. This results in increased expression of let-7e and miR-125a, as well as blocking the IL-6/STAT3 pathway, which ultimately inhibits the stemness of glioma cells and exhibits anti-tumor effects [82]. lncRNA BC200 RNA, through its regulation of the tumor suppressor miR-218-5p, inhibits the stemness of gliomas both in vitro and in vivo [83]. lncRNA FOXD2-AS1, TATA-Box binding protein-associated factor 1 (TAF-1), and Notch receptor 1 (NOTCH1) were found to be significantly upregulated in glioma tissues and GSCs. FOXD2-AS1 overexpression recruits TAF-1 to the promoter region of NOTCH1, resulting in upregulation of NOTCH1 and promotion of the stemness of gliomas [84]. Real-time quantitative polymerase chain reaction (RT-qPCR) analysis revealed high expression levels of lncRNA linc00265 and lysosomal thiol reductase (IFI30) in glioma cells. Furthermore, it was confirmed that linc00265 enhances the level of ZNF384 by sequestering miR-let-7d-5p and recruiting IGF2BP2. ZNF384, being a transcription factor of IFI30, then promotes the expression of IFI30, ultimately leading to the enhancement of glioma stemness [85].

### The regulation of lncRNAs in glioma drug resistance

**Fig. 1:**
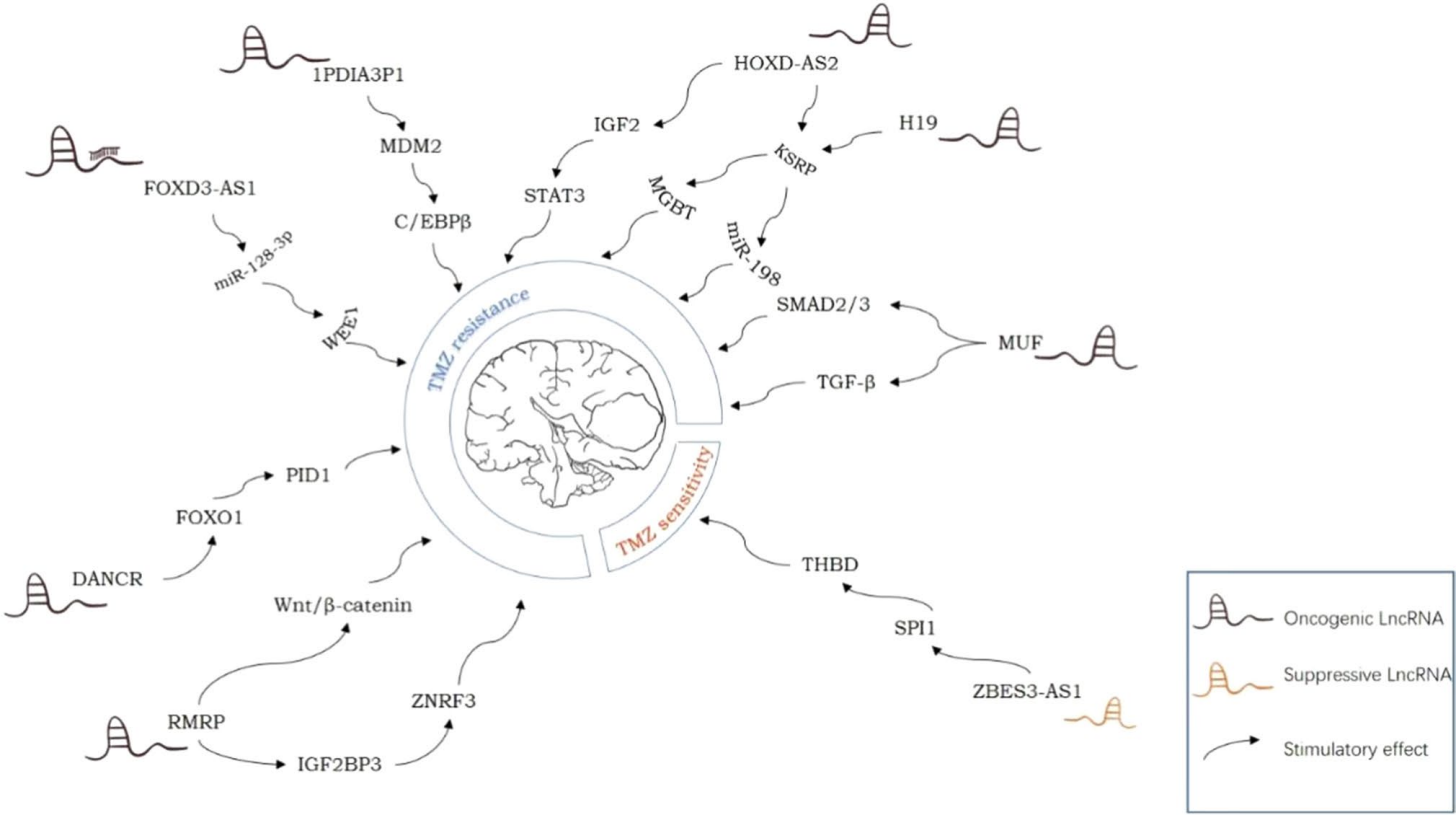
LncRNA and drug resistance in glioma

For glioma, the expression of LncRNA is closely related to chemical tolerance. Abnormal expression of various LncRNAs can be detected in drug-resistant glioma or related cell lines [86]. Numerous studies have shown that abnormally expressed lncRNAs participate in the formation of glioma drug resistance through multiple signaling pathways or other pathways (Fig. 1). For example, using microarray technology to detect the expression of LncRNA-MUF in GBM, it was found that the expression of LncRNA-MUF was significantly upregulated, and it was confirmed that the high expression of lncRNA MUF enhanced the phosphorylation of SMAD2/3 downstream of the TGF-β pathway and the gene expression of TGF-b target gene subgroups in cis and trans, thereby enhancing the resistance of GBM to temozolomide (TMZ) [87]. Zhang et al. [88] found that lncRNAH19, HOXD-AS2, and TGF-β1 were highly expressed in glioblastoma. Further research confirmed that TGF-β1 upregulated the expression of lncRNAH19 and HOXD-AS2 through the SMAD signal. However, the overexpressed lncRNAH19 and HOXD-AS2 competitively bound with KSRP (K-homology splicing regulatory protein) to enhance the expression of MGBT (methylguanine DNA methyltransferase) and prevent KSRP from binding to primary miR-198, thereby reducing the expression of miR-198 and promoting glioma resistance to temozolomide. Additionally, lncRNAH19 and HOXD-AS2 may influence the KSRP distribution mediated by TGF-β1. Zhang et al. [89] found that LncRNA HOXD cluster antisense RNA 2 (HOXD-AS2) was mainly located in the cytoplasm in neuroglioma cells and showed significantly high expression, further confirming that LncRNA HOXD-AS2 interacted with the protein IGF2BP2 to form a complex, enhancing the expression of IGF2, thereby strengthening the STAT3 signal transduction. STAT3 can also enhance the expression of HOXD-AS2 through transcriptional activation, forming a positive feedback loop and reducing the sensitivity of glioblastoma to TMZ. Through research, it was found that LncRNA ZBED3 antisense RNA 1 (ZBED3-AS1) was downregulated in TMZ-resistant GBM tissues and cell lines (U251/TMZ and U87/TMZ), and it was experimentally verified that ZBED3-AS1 could bind to the Spi-1 proto-oncogene (SPI1) to block the transcriptional activation of platelet regulator protein (THBD) mediated by SPI1, thereby decreasing the TMZ resistance of TMZ-resistant cells [76]. Through real-time fluorescence quantitative PCR (qRT-PCR) detection of TMZ-resistant and TMZ-sensitive GBM cell lines, it was found that the pseudogene of lncRNA protein disulfide isomerase family A member 3 (PDIA3P1) was highly expressed in TMZ-resistant GBM cell lines, and it was confirmed that the overexpression of PDIA3P1 destabilized the C/EBPβ-MDM2 complex by blocking ubiquitination mediated by MDM2 and stabilized the C/EBPβ protein, thereby reducing the sensitivity of GBM cells to TMZ [90]. Ling et al. [91] found that LncRNA FOXD3 antisense RNA 1 (FOXD3-AS1) and WEE1 G2 checkpoint kinase (WEE1) were highly expressed in GBM cells, and WEE1 was positively correlated with FOXD3-AS1. Further research confirmed that FOXD3-AS1 could act as a competitive endogenous RNA (ceRNA), possibly through sequestering miR-128-3p to cause overexpression of WEE1, thereby increasing the tolerance of TMZ-sensitive cells to TMZ. Liu et al. [92] found that LncRNA RMRP was significantly upregulated in neuroglioma tissues, and it was confirmed that RMRP negatively regulated the expression of ZNRF3 by IGF2BP3 in neuroglioma and decreased the stability of ZNRF3 mRNA, enhancing the resistance of neuroglioma to TMZ. In addition, the RMRP/ZNRF3 axis and the Wnt/β-catenin signaling pathway can form a positive feedback loop to regulate the resistance of TMZ in neuroglioma. Zhao et al. [93] found that LncRNA CRNDE was highly expressed in TMZ-resistant patients, and functional tests confirmed that the overexpression of CRNDE reduced the activation of the PI3K/Akt/mTOR signal, increased the expression of LC3 II/I, Beclin1, and Atg5, reduced the expression level of p62 to increase autophagy and reduce sensitivity to TMZ. A study found that LncRNA DANCR could target FOXO1 and cause its low expression, further inhibiting the binding of FOXD3-AS1 to the promoter region of PID1 to decrease the expression of PID1 protein, thereby promoting the tolerance of GBM cells to temozolomide [94].

## The potential clinical significance of lncRNAs in the occurrence and development of glioma

### lncRNAs as diagnostic biomarkers

Glioma is one of the most malignant primary tumors in the brain. However, due to the lack of effective means for diagnosing and treating glioma, the prognosis for glioma patients remains very poor. As research on LncRNA in glioma continues to deepen, it has been found to have unique expression patterns in various types of tumors and to serve as either a tumor suppressor or promoter. The expression of various LncRNAs provides important diagnostic value for glioma [2, 95]. For example, a study using LncRNA expression profiling microarrays examined the LncRNA profiles of three glioma specimens and found that 4858 LncRNAs were abnormally expressed in glioma tissues, with 2845 upregulated and 2013 downregulated. Therefore, LncRNA can serve as a biological marker to provide important evidence for glioma diagnosis [96]. Sun et al. [97] found that levels of LncRNA-ANRIL and SOX9 in the serum of glioma patients were significantly higher than in healthy individuals, and high expression of LncRNA-ANRIL and SOX9 was closely associated with poor prognosis, providing important value for diagnosing different grades of glioma. Xie et al. [98] discovered a relationship between LncRNA-TRHDE-AS1 and tumor prognosis through pan-cancer analysis. They subsequently compared the expression levels of TRHDE-AS1 in different clinical types of glioma and found significant differences in pathological typing, WHO typing, molecular typing, IDH mutation, age stratification, etc., indicating that LncRNA-TRHDE-AS1 can serve as a potential biological marker for glioma diagnosis. Zhao et al. [99] found that cancer-associated fibroblasts upregulate LncRNA DLEU1, which acts as an oncogene by binding with ZFP36 to epigenetically downregulate ATF3 expression, promoting iron death resistance in GBM, thereby providing a potential biological marker for diagnosing iron death resistance in GBM.

### lncRNAs as novel therapeutic targets

**Fig. 2:**
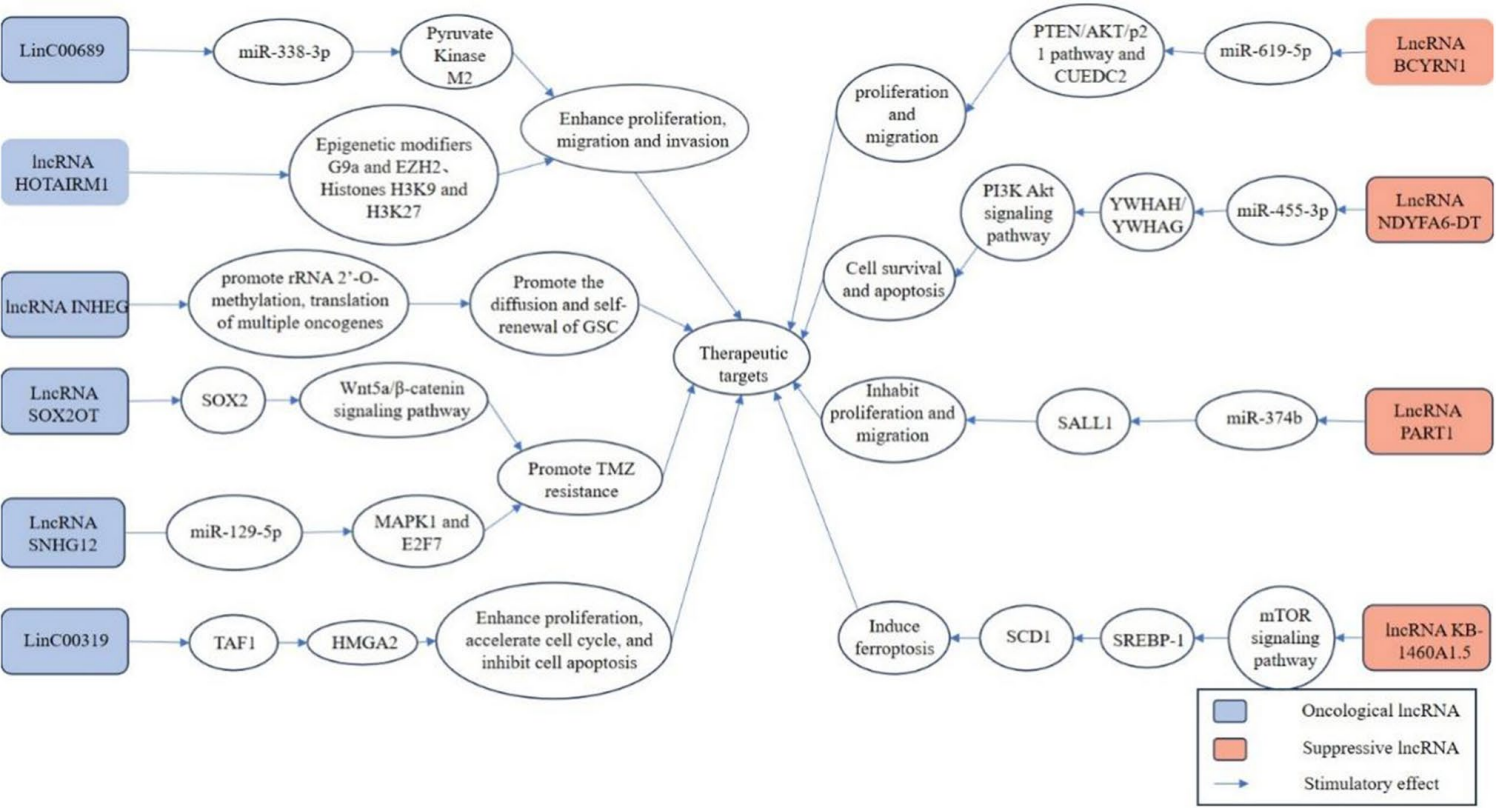
lncRNA as a potential therapeutic target for glioma treatment

For glioma, despite significant advances in therapeutic interventions, it remains one of the most deadly and invasive malignant tumors in the central nervous system, and a major cause of cancer-related mortality, with poor prognosis for patients [100]. Current treatments mostly fail because these patients almost all relapse after surgical resection and are unresponsive to therapies due to multi-drug resistance [101–103]. Numerous latest studies have shown that LncRNAs can affect the occurrence, progression, metastasis, as well as drug resistance of glioma, and play a crucial role in these processes (Fig. 2).

It has been previously discovered that oncogenic lncRNAs are overexpressed or upregulated in glioma, promoting tumor cell proliferation, invasion, migration, angiogenesis, etc., and exerting pro-tumor effects. This suggests that targeting oncogenic lncRNAs to reduce their expression or targeting their related pathways to inhibit their biological effects could have anti-tumor effects. For example, Liu et al. [73] demonstrated through functional experiments that LinC00689 promotes the expression of pyruvate kinase M2 (PKM2) by directly interacting with miR-338-3p, acting as a competitive endogenous RNA (ceRNA) to enhance glioma cell proliferation, migration, invasion, and glycolysis, which may offer new therapeutic targets and approaches for glioma treatment. Li et al. [104] found that lncRNA HOTAIRM1 mediates the demethylation of histone H3K9 and H3K27, reducing DNA methylation levels by sequestering epigenetic modifiers G9a and EZH2, and potentially affecting GBM cell proliferation, migration, invasion, and apoptosis suppression, providing a new approach for targeting HOTAIRM1 in GBM treatment. Liu et al. [105] discovered that the expression of lncRNA INHEG is significantly increased in GSCs. lncRNA INHEG promotes rRNA 2ʹ-*O*-methylation guided by snoRNPs, translation of multiple oncogenes, and enhances TAF15-NOP58 interaction and C/D box RNP assembly, thereby promoting self-renewal and spread of GSCs, which potentially offeres strategies for GBM treatment. LncRNA SOX2OT recruits ALKBH5 to demethylate the SOX2 transcript, leading to upregulation of SOX2 expression to suppress apoptosis, and activates the Wnt5a/β-catenin signaling pathway to promote cell proliferation and TMZ resistance, providing new therapeutic targets for TMZ treatment [106]. Lu et al. [107] discovered that LncRNA SNHG12 sponges miR-129-5p, leading to upregulation of MAPK1 and E2F7, conferring TMZ resistance to GBM cells, and potentially serving as a promising therapeutic target to overcome TMZ resistance and improve the clinical efficacy of TMZ chemotherapy. Further study has found that LinC00319 can directly bind to TATA box binding protein related factor 1 (TAF1) and further regulate HMGA2 to promote glioma cell proliferation, accelerate cell cycle, and inhibit glioma cell apoptosis, providing new ideas for molecular targeted therapy of glioma [108].

Many studies have found that compared to normal brain tissue, many tumor-suppressive lncRNAs are underexpressed in glioma. However, underexpressed lncRNAs may not exert effective anti-tumor effects. Therefore, enhancing the re-expression of these tumor-suppressive lncRNAs may provide new therapeutic targets for glioma treatment. For instance, Mu et al. [109] found that LncRNA BCYRN1 is significantly downregulated in glioma, and its low expression possibly positively correlates with the progression of glioma. High expression of BCYRN1 acts as a ceRNA, sponging miR-619-5p to regulate the expression of CUE domain-containing protein 2 (CUEDC2) and the PTEN/AKT/p21 pathway, ultimately inhibiting cell proliferation and migration of glioma cell lines, which offers new insights for glioma treatment. LncRNA NDYFA6-DT was found to be significantly downregulated in glioma cells, with lower levels of expression as the grade of glioma increases. Further research indicated that LncRNA NDYFA6-DT may act as a ceRNA by sponging miR-455-3p to affect downstream YWHAH/YWHAG expression levels, thereby regulating the PI3K-Akt signaling pathway and influencing the progression of Glioma [110]. Deng et al. [111] found that LncRNA PART1 and SALL1 are underexpressed, while miR-374b is upregulated in glioma, and confirmed that LncRNA PART1 targeted miR-374b to promote SALL1 expression, which inhibits glioma cell proliferation and migration. Studies have also shown that lncRNA KB-1460A1.5 acts as a tumor-suppressive lncRNA by inhibiting the mTOR signaling pathway, reducing the expression of downstream sterol regulatory element-binding protein 1 (SREBP-1), thereby attenuating the desaturation of polyunsaturated fatty acids mediated by stearoyl-CoA desaturase-1 (SCD1), inducing cancer cell oxidative stress and iron death, exerting anti-tumor effects, and having therapeutic potential [112].

### lncRNAs as prognostic biomarkers for glioma patients

Glioma is one of the most deadly malignant brain tumors in adults, with strong invasiveness and poor prognosis [113]. Surgical resection is the main treatment method for the majority of glioma, however, tumor recurrence is almost inevitable [114]. Certain clinical prognostic factors (such as age, gender, immunohistochemical subtypes, infiltration site, tumor size, EOR, BMI, ADC value, ECOG score, etc.) and biological prognostic factors (such as ki-67, IDH1, IDH2, MGMT, etc.) have been shown to predict the risk of recurrence in glioma patients [115–118]. Zhang et al. [119] identified through comprehensive clinical genomic analysis that characteristics of high-risk glioma patients with shortened survival include frequent copy number alterations, such as PTEN, CDKN2A/B deletions, EGFR amplification, fewer mutations in the IDH1 or CIC genes, high levels of immune-suppressive cell infiltration, activation of the G2M checkpoint, and oncogenic pathways involving oxidative phosphorylation. Although there are many clinical and biological prognostic factors, there is currently no clear evidence of which independent factor can be used to judge the prognosis of glioma.

With the discovery of a large number of LncRNA studies, LncRNA has gradually become an important potential target for predicting the prognosis of gliomas [120]. Through the TCAG database, it can be understood that under the WHO classification, LncRNAs show differential expression in different tissues, providing important insights for predicting the prognosis of gliomas. For example, Zhou et al. [121] demonstrated that the expression level of LncRNA ADAMTS9-AS1 is closely related to tumor size and WHO grading in a study, and through Kaplan–Meier analysis and Cox multivariate analysis, ADAMTS9-AS1 was shown to be an independent prognostic factor affecting the overall survival of glioma patients. Duan et al. [63] constructed a 15-LncRNA (including AC010173.1, HOXA-AS2, etc.) feature related to the TGF-β signaling through Cox and LASSO regression analysis, and found that this feature is a reliable prognostic tool, with high risk indicating poor prognosis, and being related to malignant clinicopathological and genomic mutation traits. Huang et al. [122] identified ten differential expression (DE-) RNA methylation-related LncRNAs through differential expression analysis and weighted gene co-expression network analysis (WGCNA), to construct prognostic features of gliomas. They found that the overall survival of high-risk groups was lower than that of low-risk groups, and the risk group informed the immune function, immune therapy response, and drug sensitivity of glioma patients in different subgroups. In addition, it can be understood through previous studies that amino acid reprogramming is an emerging hallmark of cancer [123], so analyzing amino acid-related LncRNAs can also provide important potential value for predicting the prognosis of glioma patients. For example, Lei et al. [124] identified 8 amino acid-related LncRNAs (including AL357060.1, HOXA-AS3, Linc01561, Z95115.1, AL353796.1, LEF1-AS1, AC005224.3, and TMEM220-AS1) with high prognostic value for glioma through Cox and LASSO regression analysis. They found that compared to the low-risk score group, the high-risk score group had significantly worse prognosis, more clinical pathological characteristics, and characteristic genomic distortions, and in vitro experiments showed that siRNA-mediated Linc01561 could promote the development of glioma cells, therefore providing important potential targets for predicting the prognosis of glioma patients. Research has also found that differential expression of LncRNAs can lead to differences in treatment sensitivity. For example, Li et al. [125] reported the differential expression profile of m6A-modified LncRNAs in human GBM tissues, among which WEE2-AS1 was identified as a new type of m6A-modified LncRNA, which can promote the progression of glioma stem cells and reduce the sensitivity to dasatinib, providing a potential biological marker for predicting the treatment effect of glioma patients.

## Discussion

Glioma is one of the most deadly malignant brain tumors in adults, with strong invasiveness and poor prognosis. Although progress has been made in improving surgical techniques and clinical treatment strategies, the treatment of high-grade glioma remains challenging, with low success rates, low overall survival rates, and high recurrence rates. Therefore, finding new targets and new drugs for glioma treatment is urgent. As research on LncRNA in cancer continues to deepen, it has been found to play an indispensable role in the occurrence and development of cancer. In this review, we summarize how LncRNA regulate cell proliferation, apoptosis, migration, invasion, immune response, glycolysis, angiogenesis, and drug resistance at the transcription, translation, and post-translation levels, providing important clues for the diagnosis, treatment, and prognosis of glioma. LncRNA as a biological marker and therapeutic target helps provide individualized treatment for patients, inhibit tumor growth and improve patient prognosis. However, current research on LncRNA is not comprehensive, clinical data is limited, and mostly based on small sample sizes. It is believed that further research on LncRNA in clinical settings in the future will bring new hope to the field of glioma treatment. By fully utilizing the role of LncRNA in glioma, early, rapid, and accurate diagnosis can be provided to patients, as well as personalized treatment plans. It is necessary to have a more comprehensive understanding of the function and degree of each LncRNA, invent fast and economically accurate LncRNA measurement methods, design comprehensive scoring tables to determine prognosis and treatment methods after measurement, and develop targeted drugs, all of which can bring significant benefits to patients, reduce disease pain and family burden. Furthermore, attention should be paid to the role of LncRNA in refractory glioma, in order to improve the prognosis and treatment outcomes for this group of individuals.

## Data Availability

No datasets were generated or analysed during the current study.
